# Bone morphology and alignment features are associated with knee kinematics in healthy individuals: A scoping review

**DOI:** 10.1002/jeo2.70619

**Published:** 2026-01-19

**Authors:** Erin Teule, Sebastiaan van de Groes, Nico Verdonschot, Dennis Janssen

**Affiliations:** ^1^ Orthopaedic Research Laboratory Radboud University Medical Center Nijmegen the Netherlands; ^2^ Department of Plastic Surgery Radboud University Medical Center Nijmegen the Netherlands; ^3^ Laboratory of Biomechanical Engineering University of Twente Enschede the Netherlands

**Keywords:** bone morphology, healthy knees, knee alignment, knee kinematics

## Abstract

**Purpose:**

The aim of this scoping review was to compose an overview of existing literature on the influence of knee bone morphology and alignment on knee kinematics in healthy individuals.

**Methods:**

This review was conducted according to the methodological frameworks of Arksey and O'Malley and Levac et al. and reported in accordance with the Preferred Reporting Items for Systematic Reviews and Meta‐Analyses extension for Scoping Reviews guidelines. A systematic PubMed search was performed to identify studies examining associations between knee bone morphology or alignment features and knee kinematics during movement in healthy individuals. Data were charted using a standardised form and categorised and summarised by individual bone (femur, tibia or patella) or as alignment feature.

**Results:**

A total of 2402 studies were initially identified. Following duplicate removal and eligibility screening, 29 studies were included. Knee kinematics were assessed using various techniques, including marker‐based motion capture systems and advanced dynamic magnetic resonance imaging (MRI) or computed tomography (CT) imaging, across diverse knee movement tasks. Thirteen femoral and seven tibial bone morphology features demonstrated statistically significant associations with knee kinematics, whereas only two patellar bone morphology features were statistically significantly associated. Additionally, sixteen different alignment features showed statistically significant associations with knee kinematics. The features most frequently associated with knee kinematics included lateral trochlear inclination, tibial slope, tibial tuberosity–trochlear groove distance and static patellar tilt angle.

**Conclusion:**

Although many reported associations were supported by only a limited number of studies, this review provides a comprehensive overview of relationships between knee morphology and kinematics in healthy individuals. The findings highlight the importance of considering bone morphology and alignment in kinematic assessment and contribute to a growing understanding of functional knee anatomy. The influence of patellar bone morphology on knee kinematics remains underinvestigated, underscoring the need for standardised, large‐scale studies to further advance clinical assessment and biomechanical understanding.

**Level of Evidence:**

Level III.

**Abbreviations**
AbAd: abduction–adduction
AP: anterior–posterior
IE: internal–external
LTI: lateral trochlear inclination
LTS: lateral posterior tibial slope
MTS: medial posterior tibial slope
NWB: non‐weight‐bearing
PD: proximal‐distal
PF: patellofemoral
PRISMA‐ScR: Preferred Reporting Items for Systematic Reviews and Meta‐Analyses extension for Scoping Reviews
TF: tibiofemoral
TT‐TG: tibial tuberosity–trochlear groove
WB: weight‐bearing

## INTRODUCTION

Every step we take depends on the optimal coordination of knee joint structures, underscoring the crucial role this joint plays in everyday activities [7]. Optimal knee function, arising from the complex interplay between osseous anatomy and the surrounding joint capsule, muscles, ligaments and menisci, is essential for both stability and mobility of the knee [7, 18]. A key aspect of this function is kinematics: the three‐dimensional motion of the femur, tibia and patella during knee movement. However, even among healthy populations, substantial variability in knee kinematics has been observed [9, 15, 52], challenging clinicians and researchers attempting to define normal knee kinematics.

A potential explanation for this variability lies in individual differences in knee bone morphology and alignment, as studies have shown that groups with distinct morphology or alignment features exhibit different motion patterns. For instance, healthy females with genu valgum demonstrate increased knee abduction and external rotation during motion [3]. Similarly, Martelli et al. reported that gait kinematics are influenced by tibiofemoral geometry; however, the specific geometry features driving this effect were not investigated [32]. In clinical practice, the association between anatomical shape and kinematics is still poorly understood, and observed motion patterns are not always directly attributable to the underlying anatomical geometry. As personalised treatment strategies become increasingly important, an improved understanding of how specific morphological or alignment features affect knee kinematics is increasingly required [16, 26]. Such insights could enhance the understanding of form‐function relationships in both healthy and pathological knees, thereby supporting the development of personalised diagnostic and therapeutic approaches.

Several studies have explored the relationship between knee morphology or alignment and kinematics. However, differences in study populations (e.g., cadaveric specimens, healthy or injured populations) [10, 19, 24, 28, 38] and analytical approaches, often involving complex statistical models that are difficult to interpret anatomically [12, 30, 43], hinder direct comparison and limit generalisable conclusions. To date, only one structured review has synthesised the impact of morphological differences on knee function following anterior cruciate ligament injury [27], and no comparable overview exists for healthy knees. This gap impedes the understanding of how the full spectrum of knee shape, including femoral, tibial and patellar morphology, and knee alignment features, influences kinematic outcomes in healthy knees, despite the hypothesis that specific morphological or alignment features are related to healthy knee kinematics. Therefore, the aim of this scoping review is to systematically compose an overview of the existing literature on the influence of knee bone morphology and alignment on knee kinematics in healthy individuals.

## METHODS

This scoping review was conducted following the methodological frameworks outlined by Arksey and O'Malley and Levac et al. [2, 29], and in accordance with the Joanna Briggs Institute (JBI) methodology for scoping reviews [40]. The review was designed and performed in line with the Preferred Reporting Items for Systematic Reviews and Meta‐Analyses extension for Scoping Reviews (PRISMA‐ScR) guidelines [46].

### Search strategy and study eligibility

To ensure the inclusion of relevant publications, several inclusion criteria were defined to guide the search strategy. Only primary research studies written in English or Dutch were considered, regardless of the study design. The most important inclusion criterion was that the study analysed an association between knee bone morphology or alignment features and knee kinematics in healthy knees. Alignment features, such as tibial tuberosity–trochlear groove (TT–TG) distance, are typically measured from imaging in full knee extension and reflect joint orientation rather than bone shape. These parameters were considered separately from morphological features, which describe the geometry of the bone itself. Based on these criteria, a comprehensive search strategy was developed through extensive consultation with a multidisciplinary team—including a research librarian, orthopaedic surgeon, technical physician and biomechanical engineers. The final search was executed in PubMed on 1 May 2025. All identified articles were screened using Rayyan [39]. Exclusion criteria involved non‐original studies (e.g., reviews, editorial commentaries, conference abstracts), cadaver or animal studies, studies analysing only non‐healthy knees, studies that did not analyse knee kinematics during knee motion and computational simulation studies. Although reviews were not included, studies cited within these reviews were also considered for inclusion. Studies that met the criteria were included for full‐text screening and data extraction.

### Search string for PubMed

(‘Knee Joint’[Mesh] OR ‘Knee’[Mesh] OR Knee*[Title/Abstract] OR Patella*[Title/Abstract] OR Tibiofemoral[Title/Abstract] OR Patellofemoral[Title/Abstract]) AND (‘Biomechanical Phenomena’[Mesh] OR Biomechanic*[Title/Abstract] OR Kinematic*[Title/Abstract] OR Track*[Title/Abstract] OR align*[Title/Abstract] OR rotat*[Title/Abstract] OR translat*[Title/Abstract]) AND (Morpholog*[Title/Abstract] OR Shape*[Title/Abstract] OR Anatom*[Title/Abstract] OR Geometr*[Title/Abstract] OR slope[Title/Abstract] OR distance[Title/Abstract] OR depth[Title/Abstract] OR trochlea*[Title/Abstract]) AND (Relation*[Title] OR Correlat*[Title] OR Associat*[Title] OR Influenc*[Title] OR Effect*[Title] OR Affect*[Title] OR Impact*[Title] OR Predict*[Title]) AND (English[lang] OR Dutch[lang]).

### Data charting

A standardised charting form was developed by the full research team to determine which variables were extracted from the included studies. Extracted data included author(s), year of publication, study population characteristics (sample size, age, gender), examined knee movement(s), technique used to assess knee kinematics, evaluated bone morphology or alignment features, assessed kinematic features and main findings in terms of the analysed associations and whether these were statistically significant (positive or negative association) or non‐significant. To facilitate interpretation, the identified features were categorised and summarised either by individual knee bone (femur, tibia or patella) or as knee alignment feature. Study screening, selection and data charting were performed by one reviewer (E. T.); any ambiguous cases were discussed with the full research team.

## RESULTS

**Figure 1:**
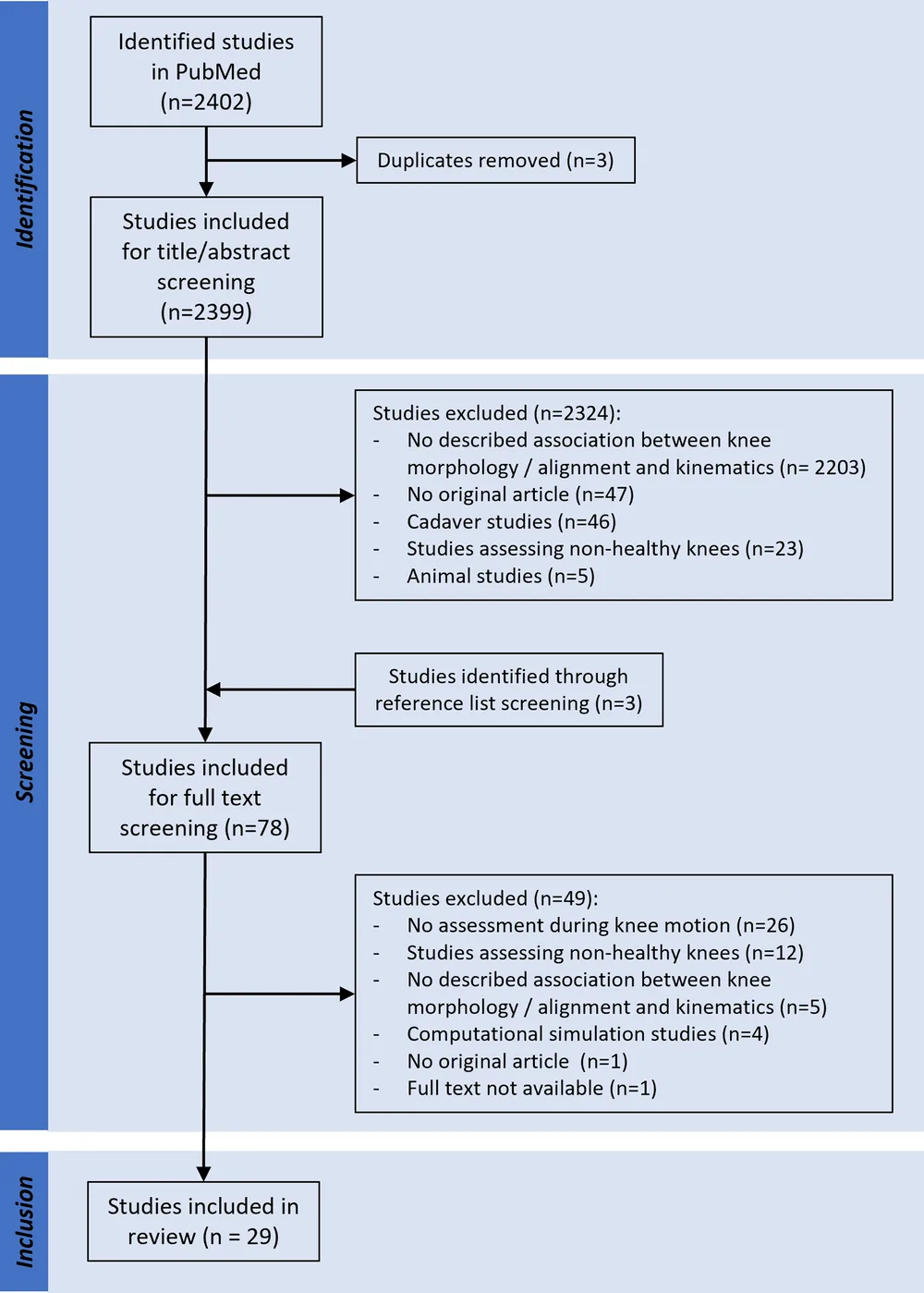
Flowchart describing the inclusion process of all articles.

The initial PubMed literature search resulted in 2402 studies. After removal of duplicates (*n* = 3), 2399 studies were screened for eligibility based on title and abstract. Most studies were excluded for not addressing an association between knee morphology or alignment features and kinematics (Figure 1). Seventy‐five studies were selected for full‐text analysis, and three additional relevant studies were identified through reference list screening, resulting in 78 full‐text studies being assessed for eligibility. Of these, 49 were excluded, primarily because they did not evaluate an association during knee motion, leaving 29 studies included in this review (Figure 1, Table 1). The included studies were published between 2000 and 2024 and had sample sizes ranging between 10 and 279 healthy participants per study. Different analysis techniques were used to assess both tibiofemoral (TF) and patellofemoral (PF) kinematics, ranging from marker‐based motion capture systems to advanced dynamic magnetic resonance imaging (MRI) or computed tomography (CT) analysis. Knee kinematics were assessed during different knee movements, including active knee flexion/extension (both weight‐bearing [WB] and non‐weight‐bearing [NWB]), gait, running, jump landing, squatting or lunge movements. All associations with at least one reported statistically significant finding are discussed in this review. A complete list of analysed associations, including the non‐significant findings, is available in Supporting Information S1.

**Table 1:** Overview of included studies.

Authors	Year	*N*	Age	Gender	Knee morphology/alignment features	Kinematic features	Examined movement(s)	Analysis technique
Arai et al. [1]	2013	28	21.5 (SD 2.7)	All F	Posterior tibial slope difference (lateral–medial)	‘Knee‐in’ angle, varus angle, flexion angle, tibial rotation at initial contact, peak knee‐in moment, peak knee‐in variation from initial contact to peak knee‐in	Single‐drop‐landing	MRI in maximum external and internal rotation + VICON motion analysis system
Bates et al. [4]	2014	239	13.6 (SD 1.6)	All F	Tibia length	Knee flexion, abduction and internal angle at initial contact; maximum abduction, adduction, internal, external, flexion and minimum flexion angle; and flexion, abduction and internal rotation ranges of motion	Double‐leg drop‐vertical jump	Motion marker analysis system (Eagle cameras)
Buzzatti et al. [5]	2024	21	29.0 (SD 11.0)	9 F, 12 M	Sulcus angle, sulcus depth, lateral trochlear inclination, TT–TG	Bisect offset, lateral patellar tilt	WB knee flexion/extension (0°−30°)	Bilateral dynamic CT imaging
Carlson et al. [6]	2017	32	28 (SD 7.9)	28 F, 10 M	TT–TG	Lateral patellar displacement	NWB knee flexion/extension (0°−45°)	Static + dynamic MRI
Clément et al. [8]	2018	90	34.8 (SD 12.3)	49 F, 41 M	Hip–knee–ankle angle	TF abduction/adduction rotation	Gait	Full‐length WB X‐ray + KneeKG system
Fick et al. [11]	2020	20	13.6 (SD 1.3)	All F	Lateral trochlear inclination, patella‐height ratio, lateral shaft length, sulcus depth, Wiberg index	PF ML shift, PF tilt	NWB knee flexion/extension	Static + dynamic MRI
Freedman et al. [14]	2013	26	25.3 (SD 7.7)	19 F, 7 M	Patellar tilt angle, lateral patellar displacement, anterior–posterior patellar displacement, inferior–superior patellar displacement, bisect offset, patellophyseal index, RF‐Q angle	Six DOF PF kinematics (measured at 10° and 20° of knee flexion)	NWB knee flexion/extension (0°−45°)	Static + dynamic MRI
Freedman et al. [13]	2014	30	25.2 (SD 7.4)	22 F, 8 M	RF‐Q angle, clinical Q‐angles: Q‐AI, Q‐AII, Q‐III	Patellar medial tilt and shift (evaluated at 10° of knee flexion)	NWB knee flexion/extension (0°−45°)	Static + dynamic MRI
Harbaugh et al. [17]	2010	33	24.9 (SD 5.1)	16 F, 17 M	Lateral trochlear inclination, sulcus angle, articular cartilage depth, sulcus groove length, trochlear bump, trochlear groove width, trochlear depth, patellar height, patellar width, facet asymmetry, condyle asymmetry, trochlear groove width: patellar width ratio	Six DOF PF kinematics, but only value at 10° and slope per kinematic parameter	NWB knee flexion/extension	Static + dynamic MRI
Hijikata et al. [20]	2023	41	68 (SD 4)	13 F, 28 M	Inclination angle of the posterior lateral and medial femoral condyles, ratio of the medial and lateral posterior condyle radii approximated as spheres, spherical condylar angle, posterior condylar angle, lateral + medial tibial slope, medial and lateral tibial slope difference, tibiofemoral rotation angle, 3D femorotibial angle, 3D hip–knee–ankle angle, passing point of the weight‐bearing line (medial–lateral and anterior–posterior)	External rotation angle of the femur relative to the tibia	Squatting	CT + WB biplane full length radiography + flat panel detector (AXIOM) & KneeMotion
Hodel et al. [21]	2022	26	27.3 (SD 10.5)	13 F, 13 M	Medial + lateral condyle width, medial + lateral condyle flexion circle, lateral femoral condyle index, medial + lateral tibial plateau slopes/depths/lengths/widths	Tibial internal/external rotation, scaled medial + lateral tibial anterior‐posterior translation	Level walking, downhill walking, stair descent	CT + biplane WB X‐ray + video fluoroscopy
Hoshino et al. [22]	2023	17	35 (SD 12)	8 F, 9 M	Condyle offset ratio, condylar twist angle	Range of anterior tibial translation; range in internal tibial rotation	Downhill running	CT + dynamic stereoradiography
Kaplan et al. [23]	2020	20	20.3 (SD 3.3)	All M	Medial + lateral tibial slope, ratio medial: lateral tibial slope, coronal tibial slope, medial tibial plateau depth	TF rotations (flexion, abduction, rotation)	Double‐legged drop landing (with load)	Standing conebeam CT + motion capture cameras with skin markers
Lynch et al. [31]	2020	61	8.6 (SD 9.54)	34 F, 27 M	Modes of shape variation obtained from statistical shape model	Six DOF TF kinematics (analysed through bivariate functional principal component analysis)	Deep kneeling (90° until full flexion)	CT + single plane fluoroscopy
Mclean et al. [33]	2010	20	21.2 (SD 1.7)	All F	Lateral + lateral posterior tibial slope, ratio medial: lateral tibial slope, tibial plateau width, intercondylar distance, ratio tibial plateau width: intercondylar distance	Knee abduction angle, knee internal rotation angle and normalised anterior knee joint reaction force	Single‐leg land‐and‐cut tasks	MRI + marker‐based kinematic analysis
McWalter et al. [34]	2010	40	28.6 (SD 8.7)	11 F, 29 M	PF kinematics at 30° of knee flexion (surrogate markers)	Six DOF PF kinematics (slope of least‐squares fit line per kinematic parameter)	WB knee flexion	MRI scans at five sequential static loaded positions between 0° and 45° knee flexion
Mozafaripour et al. [35]	2021	30	24.5 (SD 2.5)	All M	Knee hyperextension, tibial torsion, tibia vara, Q‐angle	Dynamic knee valgus	Single‐leg squat task	VICON motion analysis system
Nilstad et al. [36]	2015	279	21 (SD 4)	All F	Static knee valgus	Peak knee valgus angle	Vertical drop‐jump‐landing task	Skin marker‐based 3D motion analysis
Nukuto et al. [37]	2023	19	20.1 (SD 1.3)	8 F, 11 M	Lateral posterior tibial slope, medial tibial plateau depth, ratio lateral femoral condyle anteroposterior width: lateral tibial plateau anteroposterior width	Range of tibial internal/external rotation and range of tibial anterior–posterior translation	Fast running, double‐legged drop jump	CT + MRI + biplane radiography
Powers et al. [41]	2000	12	29.1 (SD 5.0)	All F	Sulcus angle	Bisect offset, patellar tilt angle (at 45°, 36°, 27°, 18°, 9° and 0° knee flexion)	Resisted knee extension (45°−0°) with 15% of body weight	Static + dynamic MRI MRI
Shultz et al. [42]	2012	23	21 (SD 2.8)	All F	Medial posterior tibial slope, ratio medial: lateral tibial slope, coronal tibial slope, medial tibia plateau depth	Hip adduction + internal rotation, knee valgus + internal rotation	Landing phase of double‐legged drop jumps	MRI + electromagnetic tracking with skin‐based markers (Motion Star)
Tanaka et al. [44]	2022	19	20.1 (SD 1.3)	8 F, 11 M	Medial + lateral posterior tibial slope, and medial + lateral meniscal slope	Tibial anterior–posterior translation and internal tibial rotation	Running and double‐leg drop jumps	CT + MRI + biplane radiography
Teng et al. [45]	2014	18	26.2 (SD 5.4)	All F	Sulcus angle, lateral trochlear inclination, Insall–Salvati ratio	Patellar tilt, bisect offset index	WB knee flexion (25% of body weight)	MRI (at 0°, 20°, 40° and 60° knee flexion
Varadarajan et al. [47]	2010	21	F: 29.6 (SD 10.8)M: 32.2 (SD 7.1)	11 F, 10 M	Mediolateral sulcus location, bisector angle and coronal plane angle	Patellar shift, patellar tilt, patellar rotation	Lunge task	MRI + dual plane fluoroscopic imaging
Wang et al. [48]	2024	40	55.9 (SD 7.7)	24 F, 16 M	Modified Insall–Salvati, patellar tilt angle, patella index	TF abduction/adduction rotation	Gait	Full‐length X‐ray + MRI + VICON motion system
Wang et al. [49]	2024	54	54.3 (SD 7.8)	34 F, 20 M	Q‐angle, femorotibial angle, proximal tibia varus angle, distal femoral valgus angle, sulcus angle, trochlear depth, patellar tilt angle, patellar index	TF flexion angle, extension angle, adduction angle, abduction angle, internal rotation angle, external rotation angle	Gait	X‐ray + VICON motion capture system
Ward et al. [50]	2007	13	28.1 (SD 3.9)	All F	Vertical patellar position (Insall–Salvati ratio)	Bisect offset, lateral patellar tilt angle, PF joint contact area	NWB knee flexion	MRI at 0°, 20°, 40° and 60° of knee flexion
Yuen et al. [51]	2023	10	25.0 (SD 7.7)	All F	Lateral trochlear inclination, medial trochlear inclination, sulcus angle, trochlear width, trochlear depth, condylar asymmetry, patellar width, ratio patellar width: trochlear width	Six DOF PF kinematics	Lunge task	CT + dual fluoroscopy
Zhang et al. [53]	2016	20	25 (SD 2.3)	10 F, 10 M	TT–TG, patellar tilt angle and congruence angle	Lateral patellar displacement, tibial rotation relative to femur	NWB knee flexion	Bilateral CT at 0° and 30° knee flexion

Abbreviations: 3D, three‐dimensional; CT, computed tomography; DOF, degrees of freedom; F, female; M, male; ML, mediolateral; MRI, magnetic resonance imaging; NWB, non‐weight‐bearing; PF, patellofemoral; TF, tibiofemoral; TT–TG, tibial tuberosity–trochlear groove; WB, weight‐bearing.

### Femoral bone morphology features

Eleven studies investigated associations between femoral bone morphology features and both TF and PF kinematics in healthy knees, with ten reporting thirteen features that were statistically significantly associated (Table 2) [11, 17, 20, 22, 31, 41, 45, 47, 48, 51]. One study did not report any significant associations for femoral morphology features (Supporting Information S1) [5]. Most studies analysed these associations during active knee flexion/extension [5, 11, 17, 41, 45], followed by lunge movements [47, 51], squatting [20, 31], running [22], and gait [49].

**Table 2:** Described statistically significant associations between femoral bone morphology features and knee kinematics in healthy knees.

Femoral bone morphology features	TF internal rotation	TF anterior translation	PF medial tilt	PF medial shift	PF medial rotation
Lateral trochlear inclination			+ [11, 17, 45]	+ [17, 45, 51]	
Medial trochlear inclination				− [51]	
Lateral shaft length			+ [11]		
Condyle offset ratio		+ [22]			
Condylar twist angle	+ [22]				
Sulcus angle			− [41]	− [41]	
Trochlear bisector angle			− [47]	− [47]	
Mediolateral location of the sulcus				− [47]	
Coronal plane angle of trochlear groove					+ [47]
Inclination angle of medial posterior condyle	− [20]				
Spherical condylar angle	+ [20]				
Medial condyle width	− [21]				
Lateral condyle flexion circle		+ [21]			

Abbreviations: +, a statistically significant positive association; −, a statistically significant negative association; PF, patellofemoral; TF, tibiofemoral.

### Tibial bone morphology features

Ten studies analysed relationships between tibial bone morphology features and knee kinematics in healthy knees, with nine studies reporting seven statistically significantly associated features (Table 3) [1, 4, 21, 23, 31, 33, 37, 42, 44]. One study did not report any significant association for tibial morphology features (Supporting Information S1) [20]. Interestingly, only associations between tibial features and TF kinematics were analysed, and the most frequently studied movement was jump landing [1, 4, 23, 33, 37, 42, 44], followed by running [37, 44], squatting [20, 31], and gait [21].

**Table 3:** Described statistically significant associations between tibial bone morphology features and knee kinematics in healthy knees.

Tibial bone morphology features	TF flexion	TF internal rotation	TF adduction rotation	TF anterior translation
Medial posterior tibial slope (MTS)		+ [44]		
Lateral posterior tibial slope (LTS)		+ [37, 44], − [23]		
Posterior slope difference (LTS−MTS)		+ [1]		
Posterior slope ratio (MTS:LTS)		+ [33]	− [33]	+ [21]
Coronal tibial slope			+ [42]	
Medial tibial plateau depth	− [23]	+ [37]		− [21], + [37]
Tibia length	+ [4]	+ [4]	+ [4]	

Abbreviations: +, a statistically significant positive association; −, a statistically significant negative association; PF, patellofemoral; TF, tibiofemoral.

### Patellar bone morphology features

The relationship between patellar bone morphology features and knee kinematics in healthy knees was explored in five studies, with only two studies reporting statistically significant associations in two patellar features (Table 4) [11, 51]. Three studies did not report any significant associations (Supporting Information S1) [17, 48, 49]. Patellar feature associations were studied during active knee flexion/extension [11, 17], gait analysis [48, 49] and lunge movements [51].

**Table 4:** Described statistically significant associations between patellar bone morphology features and knee kinematics in healthy knees.

Patellar bone morphology features	PF medial tilt	PF medial shift
Wiberg index	− [11]	
Lateral patellar width		+ [51]

Abbreviations: +, a statistically significant positive association; −, a statistically significant negative association; PF, patellofemoral.

### Knee alignment features

The relationship between knee alignment features and knee kinematics was examined in eighteen studies, of which twelve reported statistically significant associations between sixteen different alignment features and knee kinematics (Table 5) [5, 8, 14, 20, 33, 34, 35, 36, 37, 48, 49, 53]. Six studies did not report any significantly associated features (Supporting Information S1) [6, 11, 13, 45, 50, 51]. The most commonly analysed knee movement was active knee flexion/extension [5, 6, 8, 11, 13, 14, 34, 45, 50, 53], followed by jump landing [33, 36, 37], gait [8, 48, 49], squatting [20, 35], running [37] and lunge movements [51].

**Table 5:** Described statistically significant associations between knee alignment features and knee kinematics in healthy knees.

Knee alignment features	TF internal rotation	TF adduction rotation	TF anterior translation	PF flexion	PF medial tilt	PF medial shift	PF medial rotation	PF anterior shift	PF proximal shift
Tibial tuberosity–trochlear groove distance					− [5]	− [5, 53]			
Patellar tilt angle	− [53]	+ [48]			− [14]	− [5]			
Congruence angle	− [53]								
Patellar flexion				+ [34]					
Patellar rotation							+ [34]		
Patellar AP displacement								+ [34]	
Patellar PD displacement				+ [14]					+ [14, 34]
Patellar ML displacement					− [14]				+ [34]
Tibial external rotation angle	− [20]								
Ratio LCAP/LTAP			+ [37]						
Knee valgus angle		− [36]							
Ratio TPW/ICD		+ [33]							
Femorotibial angle		− [49]							
Hip–knee–ankle angle		− [8]							
Q‐angle		− [35, 49]							
Proximal tibia varus angle		+ [49]							

Abbreviations: +, a statistically significant positive association; −, a statistically significant negative association; AP, anterior–posterior; ICD, intercondylar distance; LCAP, lateral femoral condyle AP width; LTAP, lateral tibial plateau AP width; ML, medial–lateral; PF, patellofemoral; PD, proximal‐distal; TF, tibiofemoral; TPW, tibial plateau width.

## DISCUSSION

The most important finding of this review study was that a wide variety of morphological and alignment features of the knee are statistically significantly associated with knee kinematics in healthy individuals. Among these, lateral trochlear inclination (LTI), tibial slope, TT–TG distance and static patellar tilt angle were most frequently associated with kinematic outcomes. Overall, thirteen femoral, seven tibial, two patellar bone morphology features and sixteen alignment features were found to be statistically significantly associated with knee kinematics. However, most of these associations were reported in only one or a few studies, reflecting the heterogeneity of the current literature. Active knee flexion and extension, both WB and NWB, were identified as the most commonly examined knee movements, typically evaluated using dynamic imaging modalities such as dynamic CT or MRI. Although many studies reported statistically significant associations, a substantial number of non‐significant findings were also identified (Supporting Information S1), underscoring the complexity and variability of this research field.

### Femoral bone morphology features

Among the various femoral bone morphology features, LTI was most consistently associated with healthy knee kinematics, showing a positive association with medial patellar tilt [11, 17, 45] and medial patellar shift during both WB and NWB flexion/extension [17, 45] and lunge movements [51]. Although evidence for these associations appears strong, Buzzatti et al. could not confirm them [5]. Medial trochlear inclination was assessed in only one study, which found a statistically significant positive association with lateral patellar shift during lunging [51]. The sulcus angle has been examined in several studies, but only one reported a statistically significant association with lateral patellar tilt and shift during resisted knee extension [41], while others found no significant associations [5, 45, 51]. Other femoral morphology features have also demonstrated relevance: an association between lateral shaft length and medial patellar tilt during NWB active knee flexion/extension was reported by Fick et al. [11], and Varadarajan et al. demonstrated that the trochlear bisector angle is associated with increased lateral patellar tilt and shift, and sulcus lateralisation with increased lateral patellar shift during lunge movements [47]. This study also found a statistically significant but weak association between the trochlear coronal plane angle and lateral patellar rotation [47]. During running, Hoshino et al. found that the condyle offset ratio and condylar twist angle were positively associated with anterior tibial translation and internal tibial rotation, respectively [22]. Hijikata et al. assessed the morphology of the femoral posterior condyle during squatting and reported that the spherical condylar angle was associated with internal tibial rotation, while the inclination angle of the medial posterior condyle was associated with external tibial rotation [20]. Hodel et al. observed that medial condyle width was associated with external tibial rotation, and that the lateral condyle flexion circle was associated with anterior tibial translation during the loaded stance phase of level walking [21]. Notably, Lynch et al. was the only included study to analyse knee morphology using a statistical shape model. They found that tibiofemoral bone shape only explained up to 28% of the variation in knee kinematics during deep kneeling (90°—maximal flexion) [31]. The femoral shape features contributing to this variation, though modestly, included flattening of the distal condyles, reduced intercondylar space, altered condylar radii and bony expansions along the posterior–medial condyle and cartilage plate. These features were weakly associated with tibiofemoral internal–external (IE) rotation, anterior–posterior (AP) translation and proximal‐distal (PD) translation [31].

### Tibial bone morphology features

Most studies investigating the association between tibial bone morphology and knee kinematics focused on the posterior tibial slope and its influence on TF kinematics [1, 20, 21, 23, 33, 37, 42, 44]. However, findings regarding the association between lateral posterior slope and internal tibial rotation are mixed. While one study reported that a steeper lateral posterior slope is associated with increased internal tibial rotation [44], other studies found an association with increased external tibia rotation [23] or no significant relationship at all [20, 21, 33, 42]. One study reported that a steeper lateral posterior slope was associated with greater range of tibial IE rotation during jump landing [37]. Similarly, a steeper medial posterior slope has been associated with increased internal tibial rotation during running and jump landing [44], though other studies did not confirm this association [23, 33]. The ratio between medial and lateral posterior tibial slope has also been examined, with findings suggesting that a higher ratio (steeper medial slope) is associated with increased lateral AP translation during gait [21], as well as increased tibial internal rotation and increased tibial abduction rotation during jump landing [33]. However, these findings could not be confirmed by others [23, 42]. Arai et al. found a statistically significant positive association between posterior slope difference (lateral–medial) and tibial rotation range, though Tanaka et al. did not report such association [44]. Additionally, an increased coronal tibial slope, where the lateral side is higher than the medial, has been linked to greater tibial adduction rotation in one study [42], while another study found no signification association [23]. Collectively, these studies indicate that tibial slope influences TF kinematics, although evidence remains inconsistent. Other tibial features also demonstrated relevance: a deeper medial tibial plateau depth has been associated with decreased knee flexion [23], greater range of tibial IE rotation [37] and reduced range of tibial AP translation during jump landing [37], as well as decreased lateral anterior tibial translation during running [21]. However, Shultz and Schmitz also analysed the association between this feature and TF kinematics and reported no statistically significant associations [42]. Bates et al. found that greater tibial length was weakly but statistically significantly associated with knee flexion, tibial adduction and internal rotation during jump landing [4]. Finally, Lynch et al. used a statistical shape model to assess tibial morphology and found that variations in tibial slope and spine height, and bony expansions at the posterior–medial tibia plateau were weakly associated with tibiofemoral IE rotation, abduction–adduction (AbAd) rotation, AP translation and PD translation [31].

### Patellar bone morphology features

The influence of patellar bone morphology on knee kinematics remains insufficiently explored, with only five studies addressing this relationship, of which two reported statistically significant findings. Among the patellar morphology features studied, the Wiberg index (defined as the ratio of lateral patellar width to total patellar width) has been associated with increased lateral patellar tilt during active NWB knee flexion/extension, indicating that a wider lateral facet is associated with increased lateral tilt [11]. In contrast, two studies examining the patellar index, a similar feature, found no statistically significant associations with TF kinematics [48, 49]. Yuen et al. reported a statistically significant association between lateral patellar width and mediolateral patellar shift during lunge movement, although the direction of the shift—whether medial or lateral—was not specified [51]. Finally, the relation between patella height and PF kinematics was also investigated, but no significant associations were reported [17].

### Knee alignment features

Among the most frequently assessed alignment features are static lateral patellar tilt and TT–TG distance. Static lateral patellar tilt has been associated with decreased tibial internal rotation [53] and increased lateral patellar tilt [14] during NWB knee flexion/extension, and increased lateral patellar shift during WB knee flexion/extension [5]. Lateral patellar tilt has also been linked with increased tibial adduction during gait [48], although this was not confirmed in another study [49]. An increased TT–TG distance is associated with increased lateral patellar tilt and shift during both WB and NWB knee flexion/extension [5, 53]. Furthermore, a higher congruence angle has been associated with decreased tibial internal rotation, suggesting that patellar instability is affected by tibial rotation [53]. McWalter et al. evaluated whether static patellar kinematic outcomes at 30° of knee flexion could predict dynamic patellar motion during WB knee flexion and found statistically significant associations for patellar flexion, rotation, proximal translation and anterior translation [34]. These findings were partially supported by another study that confirmed the relationship between static and dynamic proximal translation of the patella during NWB flexion/extension [14]. McWalter et al. found no significant associations for static and dynamic patellar tilt and lateral translation [34], although Freedman et al. did report an association between static bisect offset and lateral patellar tilt [14]. Static tibial external rotation has also been linked with external tibial rotation during squatting [20]. Other assessed alignment features include the ratio between tibial plateau width and intercondylar distance, which was associated with increased tibial adduction rotation during jump landing [33]. Besides, coronal tibiofemoral alignment measures have shown associations with tibial AbAd rotation: static knee valgus angle and the hip–knee–ankle angle have been associated with tibial abduction rotation during jump landing [8, 36], and the femorotibial angle, proximal tibia varus angle, and Q‐angle have been associated with tibial AbAd rotation during gait [49]. The relationship between Q‐angle and tibial abduction was also reported during squatting [35]. Lastly, the ratio of lateral femoral condyle to lateral tibial plateau AP width has been associated with the range of anterior tibial translation during jump landing [37].

The relationship between knee shape and kinematics is multifactorial and complex; consequently, the present review has several limitations. First, the evidence supporting most identified associations is low, as the majority of the associations were investigated in only one or two studies. This restricts the ability to draw definite conclusions. Nonetheless, this review provides a comprehensive overview of the current literature. Second, there was considerable variability in the methodologies used to extract bone morphology or alignment features and to calculate kinematic data. Some studies employed automated measurements to extract morphology features, while others relied on manual techniques. Kinematic data collection also varied widely, from marker‐based motion capture systems, which are prone to soft tissue artifacts, to advanced dynamic imaging. Furthermore, kinematic calculations were based on different local coordinate systems, potentially leading to different outcomes and complicating direct comparisons between studies [25]. Imaging modalities differed as well, including (biplanar) X‐ray, CT or MRI, each yielding distinct morphology metrics and further limiting comparability between studies. Third, the included studies generally had small sample sizes (median: 29, range: 10–279), often precluding subgroup analysis and raising concerns about the generalisability of findings. Additionally, establishing associations between static and kinematic parameters proved challenging. Several studies simplified kinematic data to summary measures such as slopes or peak values. Although these simplified parameters may facilitate outcome interpretation, they may not capture the full complexity of knee motion. Furthermore, although simulation and modelling studies also explore the association between knee shape and kinematics, such studies were deliberately excluded from this review due to inherent limitations of modelling approaches, including simplifications and assumptions regarding geometry or relative motion. Nonetheless, the insights presented in this review may support future improvements in such models. Finally, although soft tissue structures play a critical role in knee function, their influence was beyond the scope of this review and was therefore not addressed in the analysis.

The concept that knee morphology and alignment features contribute to kinematic variation is reinforced by this study, suggesting a dynamic relationship between form and function in healthy knees. Although most reported associations are based on limited evidence, certain features, including LTI, tibial slope, TT–TG distance and static patellar tilt angle, emerge most consistently and appear particularly relevant for the assessment of knee kinematics. These features may serve as anatomical indicators of kinematic patterns and, if validated, could inform more precise clinical evaluation strategies. Nonetheless, the current evidence remains heterogeneous, and future research is needed to clarify these relationships, particularly regarding the underexplored influence of patellar bone morphology on knee kinematics. Large‐scale, standardised analyses will be essential to enhance the generalisability of current findings. Moreover, expanding studies to include additional contributors to kinematic variability, such as soft tissue properties and dynamic loading conditions, will be critical for capturing the full complexity of knee kinematics and advancing clinical applicability.

## CONCLUSION

This scoping review provides a comprehensive overview of the current literature on the associations between knee bone morphology and alignment features and in vivo healthy knee kinematics during movement. Morphological and alignment features most frequently associated with knee kinematics in healthy individuals include LTI, tibial slope, TT–TG distance and static patellar tilt angle. In contrast, the influence of patellar bone morphology on knee kinematics remains underexplored and warrants further investigation. While many identified associations were reported by a limited number of studies, the findings of this study contribute to a growing understanding of healthy functional knee anatomy and emphasise the importance of bone morphology in the evaluation of knee function.

## AUTHOR CONTRIBUTIONS

All authors contributed to this review. The idea came from Erin Teule, Sebastiaan van de Groes and Dennis Janssen. Erin Teule performed the literature search, data analysis and drafted the work, while Sebastiaan van de Groes, Nico Verdonschot and Dennis Janssen critically revised the work. All authors read and approved the final manuscript.

## CONFLICT OF INTEREST STATEMENT

The authors declare no conflicts of interest.

## ETHICS STATEMENT

The authors have nothing to report.

## Supporting information

Supplementary Information

## Data Availability

The authors have nothing to report.
