# Supplementary material for: Bone morphology and alignment features are associated with knee kinematics in healthy individuals: A scoping review
Source: J Exp Orthop. 2026 Jan 19;13(1):e70619. doi: 10.1002/jeo2.70619 (PMC12813864; doi:10.1002/jeo2.70619)
Supplement: Supplementary file 1 — Supplementary Information [file JEO2-13-e70619-s001.docx]

## Online Resoure 1: Analysed associations in included studies

Table 1.1: All analysed associations between femoral bone morphology features and knee kinematics in healthy knees. + denotes a statistically significant positive association, - denotes a statistically significant negative association, X denotes a non-significant association. TF = tibiofemoral; PF = patellofemoral.

| **Femoral bone morphology features** | **TF flexion** | **TF Adduction rotation** | **TF Internal rotation** | **TF Anterior translation** | **PF flexion** | **PF Medial tilt** | **PF Medial rotation** | **PF Medial shift** | **PF Anterior translation** | **PF Proximal translation** |
| --- | --- | --- | --- | --- | --- | --- | --- | --- | --- | --- |
| Sulcus depth | x ^[25]^ | X ^[25]^ | x ^[25]^ |  | x ^[27]^ | x ^[3, 6, 27]^ | x ^[27]^ | x ^[3, 6, 27]^ | x ^[27]^ | x ^[27]^ |
| Lateral trochlear inclination |  |  |  |  | x ^[9, 27]^ | + ^[6, 9, 22]^,  x ^[3, 27]^ | x ^[9, 27]^ | + ^[9, 22, 27]^ ,  x ^[3, 6]^ | x ^[9, 27]^ | x ^[9, 27]^ |
| Medial trochlear inclination |  |  |  |  | x ^[27]^ | x ^[27]^ | x ^[27]^ | - ^[27]^ | x ^[27]^ | x ^[27]^ |
| Lateral shaft length |  |  |  |  |  | + ^[6]^ |  | x ^[6]^ |  |  |
| Condyle offset ratio |  |  | x ^[12]^ | + ^[12]^ |  |  |  |  |  |  |
| Condylar twist angle |  |  | + ^[12]^ | x ^[12]^ |  |  |  |  |  |  |
| Sulcus angle | x ^[25]^ | x ^[25]^ | x ^[25]^ |  | x ^[27]^ | - ^[19]^,  x ^[3, 22, 27]^ | x ^[27]^ | - ^[19]^,  x ^[3, 22, 27]^ | x ^[27]^ | x ^[27]^ |
| Trochlear bisector angle |  |  |  |  |  | - ^[23]^ | x ^[23]^ | - ^[23]^ |  |  |
| Mediolateral location of the sulcus |  |  |  |  |  | x ^[23]^ | x ^[23]^ | - ^[23]^ |  |  |
| Coronal plane angle of trochlear groove |  |  |  |  |  | x ^[23]^ | - ^[23]^ | x ^[23]^ |  |  |
| Condylar height asymmetry |  |  |  |  | x ^[27]^ | x ^[27]^ | x ^[27]^ | x ^[27]^ | x ^[27]^ | x ^[27]^ |
| Facet asymmetry |  |  |  |  | x ^[27]^ | x ^[27]^ | x ^[27]^ | x ^[27]^ | x ^[27]^ | x ^[27]^ |
| Inclination angle of lateral posterior condyle |  |  | x ^[10]^ |  |  |  |  |  |  |  |
| Inclination angle of medial posterior condyle |  |  | - ^[10]^ |  |  |  |  |  |  |  |
| Ratio of medial/lateral posterior condyle radii |  |  | x ^[10]^ |  |  |  |  |  |  |  |
| Spherical condylar angle |  |  | + ^[10]^ |  |  |  |  |  |  |  |
| Posterior condylar angle |  |  | x ^[10]^ |  |  |  |  |  |  |  |
| Medial condyle width |  |  | - ^[11]^ | x ^[11]^ |  |  |  |  |  |  |
| Lateral condyle width |  |  | x ^[11]^ | x ^[11]^ |  |  |  |  |  |  |
| Medial condyle flexion circle |  |  | x ^[11]^ | x ^[11]^ |  |  |  |  |  |  |
| Lateral condyle flexion circle |  |  | x ^[11]^ | + ^[11]^ |  |  |  |  |  |  |
| Intercondylar distance |  | x ^[14]^ | x ^[14]^ |  |  |  |  |  |  |  |

Table 1.2: All analysed associations between tibial bone morphology features and knee kinematics in healthy knees. + denotes a significant positive association, - denotes a significant negative association, X denotes a non-significant association. TF = tibiofemoral; PF = patellofemoral.

| **Tibial bone morphology features** | **TF flexion** | **TF Adduction rotation** | **TF Internal rotation** | **TF Anterior translation** |
| --- | --- | --- | --- | --- |
| Medial posterior tibial slope (MTS) | x ^[13]^ | x ^[13, 14]^ | + ^[21]^,  x ^[10, 11, 13, 14]^ | x ^[11, 21]^ |
| Lateral posterior tibial slope (LTS) | x ^[13]^ | x ^[13, 14, 20]^ | + ^[18, 21]^, - ^[13]^,  x ^[10, 11, 14, 20]^ | x ^[11, 18, 21]^ |
| Tibial slope difference (LTS-MTS) |  | x ^[1]^ | + / x ^[1]^ , x ^[10, 21]^ | x ^[21]^ |
| Tibial slope ratio (MTS:LTS) | x ^[13]^ | - ^[14]^, x ^[13, 20]^ | + ^[14]^, x ^[11, 13, 20]^ | + ^[11]^ |
| Coronal tibial slope | x ^[13]^ | + ^[20]^, x ^[13]^ | x ^[13, 20]^ |  |
| Tibial plateau width |  | x ^[14]^ | x ^[14]^ |  |
| Medial tibial plateau depth | - ^[13]^ | x ^[13, 20]^ | + ^[18]^, x ^[11, 13, 20]^ | - ^[11, 18]^ |
| Lateral tibial plateau depth |  |  | x ^[11]^ | x ^[11]^ |
| Medial tibial plateau length |  |  | x ^[11]^ | x ^[11]^ |
| Lateral tibial plateau length |  |  | x ^[11]^ | x ^[11]^ |
| Medial tibial plateau width |  |  | x ^[11]^ | x ^[11]^ |
| Lateral tibial plateau width |  |  | x ^[11]^ | x ^[11]^ |
| Tibia length | + ^[2]^ | + ^[2]^ | + ^[2]^ |  |

Table 1.3: All analysed associations between patellar bone morphology features and knee kinematics in healthy knees. + denotes a significant positive association, - denotes a significant negative association, X denotes a non-significant association. TF = tibiofemoral; PF = patellofemoral.

| **Patellar bone morphology features** | **TF flexion** | **TF Adduction rotation** | **TF Internal rotation** | **PF flexion** | **PF Medial tilt** | **PF Medial rotation** | **PF Medial shift** | **PF Anterior translation** | **PF Proximal translation** |
| --- | --- | --- | --- | --- | --- | --- | --- | --- | --- |
| Wiberg index |  |  |  |  | - ^[6]^ |  | x ^[6]^ |  |  |
| Patella index | x ^[25]^ | x ^[24, 25]^ | x ^[25]^ |  |  |  |  |  |  |
| Lateral patellar width |  |  |  | x ^[27]^ | x ^[27]^ | x ^[27]^ | + ^[27]^ | x ^[27]^ | x ^[27]^ |
| Patella height |  |  |  | x ^[9]^ | x ^[9]^ | x ^[9]^ | x ^[9]^ | x ^[9]^ | x ^[9]^ |

Table 1.4: All analysed associations between knee alignment features and knee kinematics in healthy knees. + denotes a significant positive association, - denotes a significant negative association, X denotes a non-significant association. TF = tibiofemoral; PF = patellofemoral; AP = anteroposterior; PD = proximodistal; ML = mediolateral; LCAP = lateral femoral condyle AP width; LTAP = lateral tibial plateau AP width; TPW = tibial plateau width; ICD = intercondylar distance.

| **Knee alignment features** | **TF flexion** | **TF Adduction rotation** | **TF Internal rotation** | **TF Anterior translation** | **PF flexion** | **PF Medial tilt** | **PF Medial rotation** | **PF Medial shift** | **PF Anterior translation** | **PF Proximal translation** |
| --- | --- | --- | --- | --- | --- | --- | --- | --- | --- | --- |
| Tibial tuberosity – trochlear groove distance |  |  |  |  |  | - ^[3]^ |  | - ^[3, 28]^, x ^[4]^ |  |  |
| Patellar tilt angle | x ^[25]^ | + ^[24]^, x ^[25]^ | - ^[28]^, x ^[25]^ |  | x ^[8]^ | - ^[8]^, x ^[15]^ | x ^[8]^ | - ^[3]^, x ^[8]^ | x ^[8]^ | x ^[8]^ |
| Congruence angle |  |  | - ^[28]^ |  |  |  |  |  |  |  |
| (modified) Insall-salvati ratio |  | x ^[24]^ |  |  |  | x ^[6, 22, 26]^ |  | x ^[6, 22, 26]^ |  |  |
| Patellar flexion |  |  |  |  | + ^[15]^ |  |  |  |  |  |
| Patellar rotation |  |  |  |  |  |  | + ^[15]^ |  |  |  |
| Patellar AP displacement |  |  |  |  | x ^[8]^ | x ^[8]^ | x ^[8]^ | x ^[8]^ | x ^[8]^ | x ^[8]^ |
| Patellar PD displacement |  |  |  |  | + ^[8]^ | x ^[8]^ | x ^[8]^ | x ^[8]^ | x ^[8]^ | + ^[8, 15]^ |
| Patellar ML displacement |  |  |  |  | x ^[8]^ | - ^[8]^ | x ^[8]^ | x ^[8, 15]^ | x ^[8]^ | x ^[8]^, + ^[15]^ |
| Knee valgus angle |  | - ^[17]^ |  |  |  |  |  |  |  |  |
| Hip-Knee-Ankle angle |  | - ^[5]^ | x ^[10]^ |  |  |  |  |  |  |  |
| Ratio LCAP / LTAP |  |  | x ^[18]^ | + ^[18]^ |  |  |  |  |  |  |
| Ratio TPW / ICD |  | + ^[14]^ | x ^[14]^ |  |  |  |  |  |  |  |
| Patella-to-trochlea width ratio |  |  |  |  | x ^[27]^ | x ^[27]^ | x ^[27]^ | x ^[27]^ | x ^[27]^ | x ^[27]^ |
| Tibial external rotation angle |  |  | - ^[10]^ |  |  |  |  |  |  |  |
| Femorotibial angle | x ^[25]^ | - ^[25]^ | x ^[10, 25]^ |  |  |  |  |  |  |  |
| Passing point of WB line on tibia plateau |  |  | x ^[10]^ |  |  |  |  |  |  |  |
| Passing point of WB line on tibia plateau |  |  | x ^[10]^ |  |  |  |  |  |  |  |
| Proximal tibia varus angle | x ^[25]^ | + ^[25]^ | x ^[25]^ |  |  |  |  |  |  |  |
| Distal femoral valgus angle | x ^[25]^ | x ^[25]^ | x ^[25]^ |  |  |  |  |  |  |  |
| Knee hyperextension angle |  | x ^[16]^ |  |  |  |  |  |  |  |  |
| Tibial torsion angle |  | x ^[16]^ |  |  |  |  |  |  |  |  |
| Tibial varus angle |  | x ^[16]^ |  |  |  |  |  |  |  |  |
| Q-angle | x ^[25]^ | - ^[16, 25]^ | x ^[25]^ |  |  | x ^[7]^ |  | x ^[7]^ |  |  |
| Q-angle I (knee in full extension, quadriceps relaxed) |  |  |  |  |  | x ^[7]^ |  | x ^[7]^ |  |  |
| Q-angle II (knee in full extension, quadriceps contracted) |  |  |  |  |  | x ^[7]^ |  | x ^[7]^ |  |  |
| Q-angle III (knee in 15 deg flexion, quadriceps relaxed) |  |  |  |  |  | x ^[7]^ |  | x ^[7]^ |  |  |

References

1. Arai T, Miaki H. Influence of static alignment of the knee, range of tibial rotation and tibial plateau geometry on the dynamic alignment of "knee-in" and tibial rotation during single limb drop landing. Clin Biomech (Bristol). 2013;28:642-648.

2. Bates NA, Myer GD, Hewett TE. Prediction of kinematic and kinetic performance in a drop vertical jump with individual anthropometric factors in adolescent female athletes: implications for cadaveric investigations. Ann Biomed Eng. 2015;43:929-936.

3. Buzzatti L, Keelson B, Hereus S, Van den Broeck J, Scheerlinck T, Van Gompel G, et al. Investigating patellar motion using weight-bearing dynamic CT: normative values and morphological considerations for healthy volunteers. Eur Radiol Exp. 2024;8:106.

4. Carlson VR, Sheehan FT, Shen A, Yao L, Jackson JN, Boden BP. The Relationship of Static Tibial Tubercle-Trochlear Groove Measurement and Dynamic Patellar Tracking. Am J Sports Med. 2017;45:1856-1863.

5. Clement J, Toliopoulos P, Hagemeister N, Desmeules F, Fuentes A, Vendittoli PA. Healthy 3D knee kinematics during gait: Differences between women and men, and correlation with x-ray alignment. Gait Posture. 2018;64:198-204.

6. Fick CN, Grant C, Sheehan FT. Patellofemoral Pain in Adolescents: Understanding Patellofemoral Morphology and Its Relationship to Maltracking. Am J Sports Med 2020;48:341-350.

7. Freedman BR, Brindle TJ, Sheehan FT. Re-evaluating the functional implications of the Q-angle and its relationship to in-vivo patellofemoral kinematics. Clin Biomech (Bristol). 2014;29:1139-1145.

8. Freedman BR, Sheehan FT. Predicting three-dimensional patellofemoral kinematics from static imaging-based alignment measures. J Orthop Res. 2013;31:441-447.

9. Harbaugh CM, Wilson NA, Sheehan FT. Correlating femoral shape with patellar kinematics in patients with patellofemoral pain. J Orthop Res 2010;28:865-872.

10. Hijikata H, Tanifuji O, Mochizuki T, Sato T, Watanabe S, Katsumi R, et al. The morphology of the femoral posterior condyle affects the external rotation of the femur. J Exp Orthop. 2023;10:122.

11. Hodel S, Postolka B, Flury A, Schutz P, Taylor WR, Vlachopoulos L, Fucentese SF. Influence of Bone Morphology on In Vivo Tibio-Femoral Kinematics in Healthy Knees during Gait Activities. J Clin Med. 2022;11.

12. Hoshino Y, Wang JH, Lorenz S, Fu FH, Tashman S. The effect of distal femur bony morphology on in vivo knee translational and rotational kinematics. Knee Surg Sports Traumatol Arthrosc. 2012;20:1331-1338.

13. Kaplan JT, Ramsay JW, Cameron SE, Seymore KD, Brehler M, Thawait GK, et al. Association Between Knee Anatomic Metrics and Biomechanics for Male Soldiers Landing With Load. Am J Sports Med. 2020;48:1389-1397.

14. McLean SG, Lucey SM, Rohrer S, Brandon C. Knee joint anatomy predicts high-risk in vivo dynamic landing knee biomechanics. Clin Biomech (Bristol). 2010;25:781-788.

15. McWalter EJ, Macintyre NJ, Cibere J, Wilson DR. A single measure of patellar kinematics is an inadequate surrogate marker for patterns of three-dimensional kinematics in healthy knees. Knee. 2010;17:135-140.

16. Mozafaripour E, Seidi F, Minoonejad H, Mousavi SH, Bayattork M. Can lower extremity anatomical measures and core stability predict dynamic knee valgus in young men? J Bodyw Mov Ther. 2021;27:358-363.

17. Nilstad A, Krosshaug T, Mok KM, Bahr R, Andersen TE. Association Between Anatomical Characteristics, Knee Laxity, Muscle Strength, and Peak Knee Valgus During Vertical Drop-Jump Landings. J Orthop Sports Phys Ther. 2015;45:998-1005.

18. Nukuto K, Gale T, Yamamoto T, Musahl V, Anderst W. Bone morphology features associated with knee kinematics may not be predictive of ACL elongation during high-demand activities. Knee Surg Sports Traumatol Arthrosc. 2023;31:5096-5103.

19. Powers CM. Patellar kinematics, part II: the influence of the depth of the trochlear groove in subjects with and without patellofemoral pain. Phys Ther 2000;80:965-978.

20. Shultz SJ, Schmitz RJ. Tibial plateau geometry influences lower extremity biomechanics during landing. Am J Sports Med 2012;40:2029-2036.

21. Tanaka MJ, Elias JJ, Williams AA, Demehri S, Cosgarea AJ. Characterization of patellar maltracking using dynamic kinematic CT imaging in patients with patellar instability. Knee Surg Sports Traumatol Arthrosc 2016;24:3634-3641.

22. Teng HL, Chen YJ, Powers CM. Predictors of patellar alignment during weight bearing: an examination of patellar height and trochlear geometry. Knee 2014;21:142-146.

23. Varadarajan KM, Freiberg AA, Gill TJ, Rubash HE, Li G. Relationship between three-dimensional geometry of the trochlear groove and in vivo patellar tracking during weight-bearing knee flexion. J Biomech Eng 2010;132:061008.

24. Wang Y, Lu J, Wang Z, Li Z, Pan F, Zhang M, et al. The association between patella alignment and morphology and knee osteoarthritis. J Orthop Surg Res 2024;19:509.

25. Wang Z, Lu J, Ge H, Li Z, Zhang M, Pan F, et al. Morphology and transverse alignment of the patella have no effect on knee gait characteristics in healthy Chinese adults over the age of 40 years. Front Bioeng Biotechnol 2024;12:1319602.

26. Ward SR, Terk MR, Powers CM. Patella alta: association with patellofemoral alignment and changes in contact area during weight-bearing. J Bone Joint Surg Am 2007;89:1749-1755.

27. Yuen J, Esfandiarpour F, Lebrun CM, Dhillon S. Using Dual-Orthogonal Fluoroscopy and CT to Assess the Relationship Between Knee Morphology and Patellar Kinematics in Patients With Patellofemoral Pain. Cureus 2023;15:e44139.

28. Zhang LK, Wang XM, Niu YZ, Liu HX, Wang F. Relationship between Patellar Tracking and the "Screw-home" Mechanism of Tibiofemoral Joint. Orthop Surg 2016;8:490-495.
